# Ethnobotany of traditional cosmetics among the Oromo women in Madda Walabu District, Bale Zone, Southeastern Ethiopia

**DOI:** 10.1186/s13002-024-00673-0

**Published:** 2024-03-22

**Authors:** Siraj Sultan, Habte Telila, Lemessa Kumsa

**Affiliations:** 1 Department of Biodiversity Conservation and Ecotourism, Madda Walabu University, P.O. Box 247, Bale Robe, Ethiopia; 2 Department of Applied Biology, Adama Science and Technology University, P.O. Box 1888, Adama, Ethiopia

**Keywords:** Leaves, Maceration, Plant species, Skincare, Topical application

## Abstract

Traditional cosmetics play a significant role in the cultural practices and personal hygiene of many ethnic groups worldwide. The Oromo, an indigenous community in Ethiopia, has a rich history of utilizing local plants for cosmetic purposes*.* However, the use of plants as traditional cosmetics has only been poorly investigated as more emphasis was given to traditional medicines. The study thus aimed to identify and document plant species, and parts used in traditional cosmetics and associated knowledge, and practices among Oromo women in the Madda Walabu district, Southeastern Ethiopia. A total of 150 Oromo women were interviewed to gather ethnobotanical information including the plant species used for cosmetics, their preparation methods, and their applications. Ethnobotanical indices were computed to determine the most important species used by the women. A total of 48 plant species belonging to 31 families used as sources of traditional cosmetics were recorded. Most of these plants were trees. Leaves were the most commonly used plant part in traditional cosmetics, and maceration and decoction were the most common preparation methods applied to prepare traditional cosmetics. Topical application of traditional cosmetics was the most common, while oral infusions were rarely reported type of administration. All categories had high informant consensus factor (ICF) values, ranging from 0.80 to 0.98. The highest ICF value was found for skin treatment, followed by the hair, and face mask. Overall*, Commiphora habessinica, Gnidia stenophylla, Kalanchoe lanceolata, Mimusops kummel, Sesamothamnus rivae, Terminalia brownii, T. laxiflora, Acacia drepanolobium, A. mellifera*, and *Aloe citrina* were the most frequently cited and culturally important plants by Oromo women for traditional cosmetics in the area. The study highlights the importance of local plant resources for maintaining cultural practices and personal hygiene. However, the cultural heritage associated with traditional cosmetics is facing threats from various factors. Therefore, a continuous effort to document and disseminate knowledge about traditional cosmetics practices to ensure their preservation and transmission and awakening younger generations about the importance of traditional cosmetics and their role in cultural heritage is crucial.

## Introduction

Historical pieces of evidence revealed that human beings have been using traditional cosmetics for thousands of years (6000 BC) to enhance their natural beauty, for protection of their skin, care for teeth, and decorate their skin for cultural and religious purposes [1]. In many countries around the world, traditional societies have always used natural substances such as plant extracts for several reasons including skin protection against the sun, and skin beauty, as well as for conveying messages of tribal identity [2, 3]. Egypt was a pioneer country in the use of plants for cosmetics that goes back to the age of Pharaohs [4, 5], and the Romans, the Greeks, and the Arabs, through the medieval and Elizabethan periods and into modern times [5, 6].

The demand for beauty products such as soaps, face washes, shampoos, conditioners, skin lotions, toners, astringents, cold creams and other moisturizing formulations, perfumes, hair colors, dyes, powders, eye and face packs, etc., has been growing globally requiring trillion-dollar investment [7]. Cosmetic-pharmaceutical products were intended to improve not only the beauty but also the health of individuals [8]. On the other hand, due to their potential negative health effects in recent years, cosmetic products of synthetic origin have been widely criticized which has increased interest in the use of traditional cosmetics as they are considered less toxic, effective, and believed to contain antioxidants [9]. Most of these cosmetics were prepared from plants. Traditional cosmetics are natural cosmetics into which no synthetic chemical ingredients are added and made locally by the members of the ethnic group. Even today, traditional cosmetics are in use in many developing countries and Ethiopia is no exception. In preparing traditional cosmetics, different ethnic groups in the world have been using different species of plants. Furthermore, cross-culturally, the use of cosmetics is more common among women than men. This arises from the potential interest of women to attract their male partners and to a rival competition [10]. Moreover, the use of cosmetics makes women appear healthier, more attractive, and more feminine [8].

In any culture, the knowledge and practices of ethnobiology vary by, ethnicity, religion, profession, educational background, social status and relations, income class, age, and gender [11]. Women are likely the most important in the management of plant biodiversity in biodiversity-rich regions including Ethiopia [12, 13].

The indigenous knowledge of the preparation and use of traditional cosmetics has a long history. This knowledge has been transferred from generation to generation orally [14]. With the increasing development of technologies, however, the knowledge of these traditional cosmetics has been decreasing [9]. In addition, plant diversity from which these cosmetics are believed to be made is also depleting globally due to anthropogenic factors such as the expansion of agriculture in addition to the invisibility of the environmental benefits that are derived from women's biodiversity management [15]. Furthermore, the use of plants as traditional cosmetics has only been poorly investigated in different regions including Ethiopia [16, 17] as more emphasis was given to traditional medicines despite their important role in the economy, improvement of beauty, and minimizing health risks of synthetic cosmetics. Thus, the objective of this study was to identify and document plant species, and parts used in traditional cosmetics and associated knowledge, and practices including methods of preparation and administration among Oromo women in the Madda Walabu district.

## Materials and methods

### Study area

**Fig. 1:**
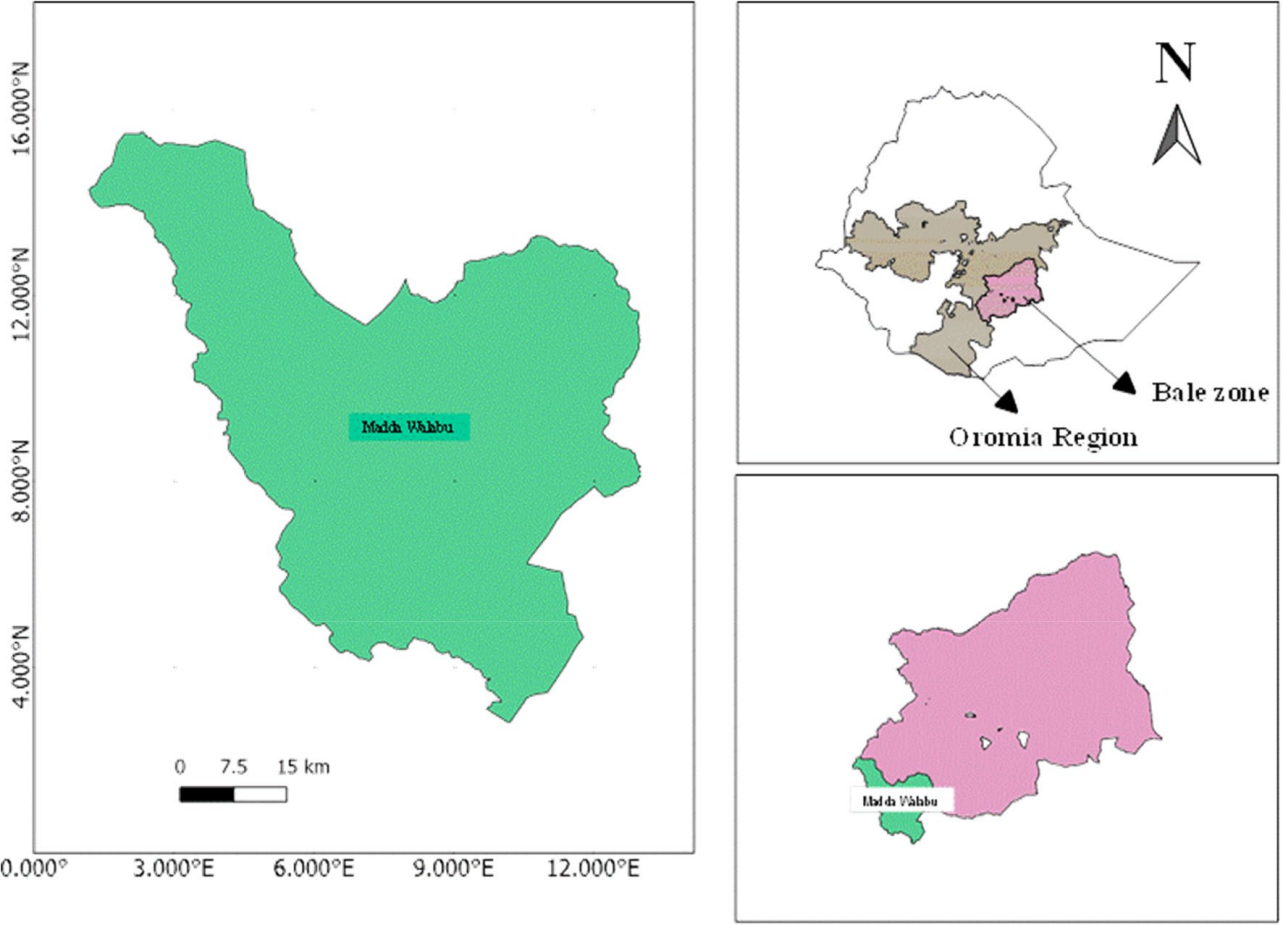
Map of the study area

The study was conducted in Madda Walabu district, Bale Zone, Oromia National Regional State, Southeastern Ethiopia, located between 9º44′32″and 9º46′26″N and 39º 44′00″and 39º 47′19″E (Fig. 1). It is located at about 630 km from Addis Ababa and 200 km from the zone capital (Robe town) in the Southeast. The majority of the inhabitants in the district are Oromo in ethnic group and Muslim in religion. The total population of the district is 140,893 (Male = 70,540 and Female = 70,353) [18]. The rainfall is bimodal, with the main rainy season occurring from early March through June and the short rain from late September through November. There are five dry months in the area, i.e., January, February, July, August, and December [19]. The most common agricultural system in the district is mixed farming with livestock and subsistence agriculture forming the major livelihoods of the rural community [20]. The vegetation type of the district is mainly *Acacia–Commiphora* woodland and Combretum–Terminalia woodlands [21].

We focused on Madda Walabu district because of its historical remark on the history of the Oromo people. The word “Madda Walabu” means Gadaa revival, the foundation of Oromo civilization and the birthplace of the Gadaa system. The Gadaa system is an ancient and indigenous democratic system of governance used by the Oromos in Ethiopia and northern Kenya) [22]. Gadaa system has been registered as one of the Intangible Cultural Heritage of Humanity by UNESCO since 2016 [23]. In Oromo, the Gadaa system is responsible for regulating political stability, economic growth, social services, cultural commitments, and the ethical contract of the religious order [24]. Oromo women had an influential position in the past. For instance, the wife of the designated Gadaa leader known as “*Abbaa Gadaa*” is equitably treated like the *Abbaa Gadaa* himself. Besides “*Siinqee”* feminists represent women in the Gadaa system, although this has now declined following the decline in the people’s indigenous cultural practices [25]. Oromo is the most dominant ethnic group in the area and is the most populous ethnic group in the country [26] accounting for approximately 40% of the currently estimated 110 million total population of the country [27].

### Data collection

A total of 150 informants (all of whom are women) were selected purposively to obtain high-quality and reliable information [28]. The selection of informants was based on the recommendations of elders and local authorities. Data collection was focused on three villages (Hora Kore, Aba Sirba, and Medacho) (Table 1) of the Madda Walabu district due to their experiences in the use of traditional cosmetics. All the respondents were living in rural areas and from a variety of socioeconomic strata, with knowledge of traditional cosmetics. Data including the local names of the plant species used for traditional cosmetics, their preparation, and administration methods were collected using semi-structured interviews from June to August 2021. Plant specimens collected during the fieldwork were identified using the Flora of Ethiopia and Eritrea. Furthermore, we used the Plants of the World Online [29] website to confirm the names of the species and their respective classification of genus and family, and finally, all voucher specimens were coded in the field, brought, and deposited in the teaching herbarium of Madda Walabu University for further investigation and later use.

**Table 1:** Population and mean altitudes (see also Appendix Table 13) of each of the three villages used to collect ethnobotanical data of traditional cosmetics among the Oromo Women in Madda Walabu District of Bale Zone, Oromia Regional State, Southeastern Ethiopia

District	Sites	Population					
		Mean Alt	Both Sexes	Male	Female	Number of households	
	Hora Kore	970	8765	4472	4293	1897	
Dallo Mana	Aba Sirba	1341	4434	2234	2200	869	
	Medacho	1256	3735	1826	1909	762	
	Total		94,543	47,539	47,004	19,638	

### Data analysis

#### Fidelity level

The level of fidelity (Fl) was computed to determine the most important plant species used by women for traditional cosmetics employing the formula used in [30].

$${\text{\% FL}} = \frac{{{\text{Ip}}}}{{{\text{Iu}}}}X100$$

where IP is the number of informants who independently suggested the use of a species for a particular use category

Iu is the total number of informants who mentioned the plant for any use category.

The more the value of FL is close to 1, the higher the number of informants that used this plant species for that particular use. This index answers the question: “Which use is associated with this particular plant?” Moreover, a one-way analysis of variance (ANOVA) was used to assess the difference in the use of traditional cosmetics among the Oromo women based on their sociodemographic features such as age, level of education, marital status, and occupation. Free *R* software was used for all the analyses [31].

### Informant consensus factor

The informant consensus factor (ICF) was calculated to assess the variability of plant usage in cosmetics within each of the ten categories using the formula used in [32]

$${\text{ICF}} = \frac{{{\text{Nur}} - {\text{Nt}}}}{{{\text{Nur}} - 1}}$$

where Nur is the number of usages reported for a category.

Nt is the number of plant species reported to be used in that particular category.

ICF values vary between 0 and 1 and a value close to 1 indicates strong consensus among informants, that is, a large proportion of the informants use the same species for the same purpose. When close to 0, the IFC value indicates a strong disagreement among informants.

### Relative frequency of citations and cultural importance index

The relative frequency of citations that do not consider the use category for traditional cosmetic plant species used by Oromo women was calculated using the following formula [33].

$${\text{RFCs}} = {\text{FCs}}/{\text{N}}$$

where RFCs = relative frequency of citations, FCs = frequency of citations, and *N* = the number of informants participating in the survey.

### Cultural importance index

The cultural importance index can be seen as the sum of the proportion of informants that mention each species use. It is an additive index used to determine the spread of the use of plant species and the diversity of its use and calculated using the formula [33]:

$${\text{CIs}} = \mathop \sum \limits_{u = ui}^{{u{\text{NC}}}} \mathop \sum \limits_{i = i1}^{iN} U{\text{Rui}}/N$$

where CIs cultural importance index, UR use report, *N* the number of informants who participated in the survey, and NC total number of use categories.

## Results

### Sociodemographic features 

#### Demographic characteristics of the informants

In the study, although the age of the participants ranged from 20 to 75 years, most of the respondents were above 50 years old. Concerning education, the majority of the participants did not attend formal education at all and attended primary education. Most of the respondents were married and housewives in occupation (Table 2).

**Table 2:** Demographic characteristics of the female respondents (i.e., Oromo women) in Madda Walabu district, Bale Zone, Southeastern Ethiopia

Demographic characteristics		Frequency	Proportion (%)
Age	< 30	15	10.00
	30 and 50	52	34.67
	> 50	83	55.33
	Total	150	100.00
Education	No formal education	70	46.67
	Primary	60	40.00
	Secondary	20	13.33
	Total	150	100.00
Marital status	Single	10	6.67
	Married	130	86.67
	Divorced	5	3.33
	Widowed	5	3.33
	Total	150	100.00
Occupation	Student	5	3.33
	Housewife	125	83.33
	Self employed	15	10.00
	Government employed	5	3.33
	Total	150	100.00

### Composition of plant species used as a traditional cosmetic in Madda Walabu district

A total of 48 plant species belonging to 39 genera and 31 families were recorded as a source of traditional cosmetics among Oromo women in the Madda Walabu District of Bale Zone (Appendix Table 10). The most common family was Fabaceae represented by six species followed by Bigonaceae, Burseraceae, and Combretaceae (Table 3; Appendix Table 10).

**Table 3:** List of plant families with their frequency

Family	Freq	Proportion	Family	Freq	Proportion
Aloaceae	1	2.08	Meliaceae	1	2.08
Anacardiaceae	1	2.08	Myrtaceae	1	2.08
Bignoniaceae	4	8.33	Olacaceae	1	2.08
Boraginaceae	3	6.25	Oleaceae	1	2.08
Burseraceae	4	8.33	Pedaliaceae	1	2.08
Capparidaceae	1	2.08	Rhamnaceae	1	2.08
Caricaceae	1	2.08	Rutaceae	2	4.17
Combretaceae	4	8.33	Salvadoraceae	1	4.17
Commelinaceae Crassulaceae	1	2.08	Santalaceae	1	2.08
	1	2.08	Sapindaceae	1	2.08
Cupressaceae	1	2.08	Sapotaceae	1	2.08
Euphorbiaceae	3	6.25	Simaroubaceae	1	2.08
Fabaceae	6	12.50	Solanaceae	2	4.17
Lamiaceae	1	2.08	Thymelaeaceae	1	2.08
Lauraceae	1	2.08	Tiliaceae	1	2.04
Loganiaceae	1	2.08			

### Traditional cosmetics

#### Plant parts and growth forms of traditional cosmetics

**Fig. 2:**
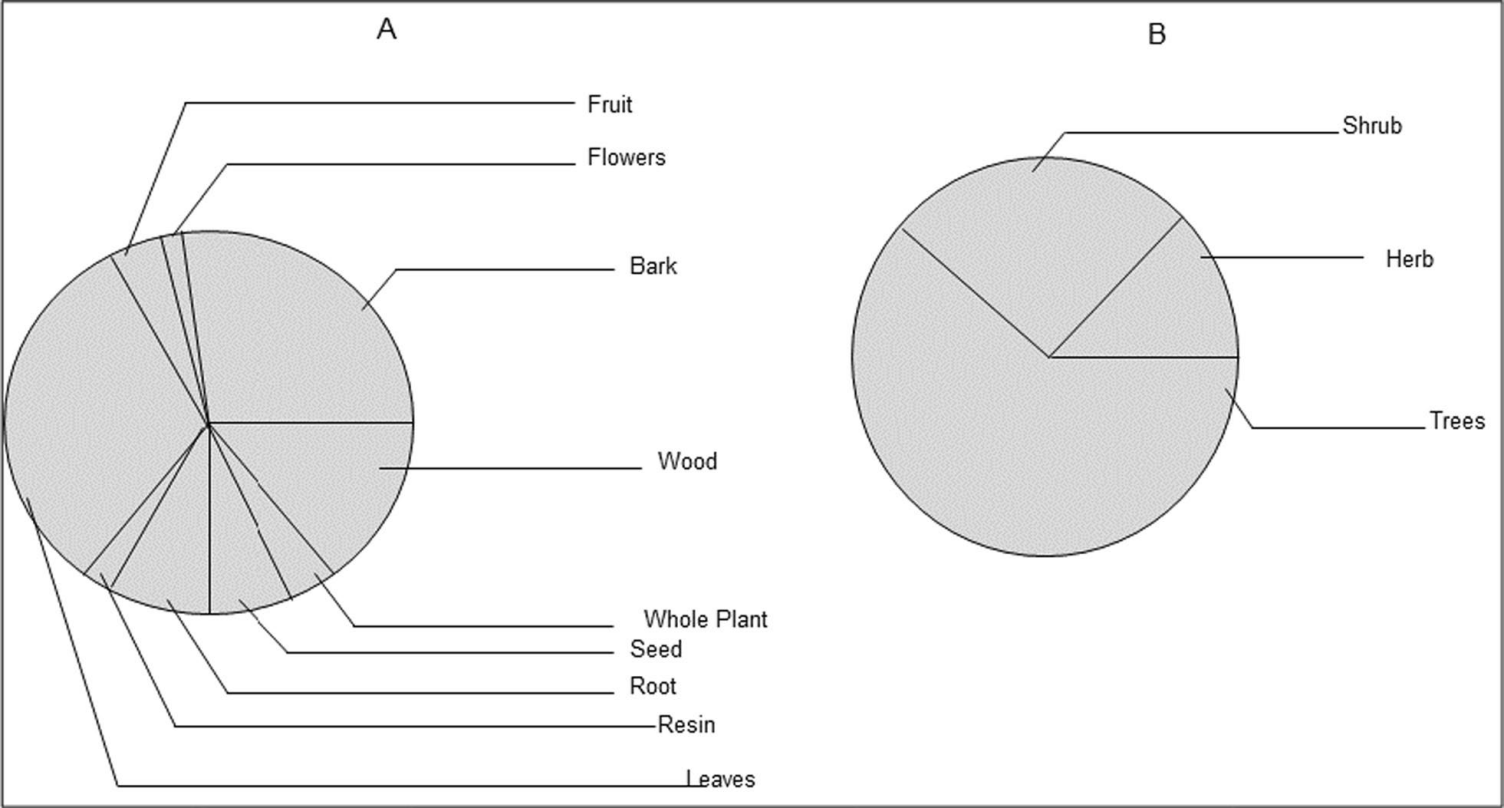
Proportion of **A** plants parts, **B** growth forms of plant species used as a traditional cosmetic among the Oromo women in the study area

The most common plant parts used in the preparation of traditional cosmetics were leaves followed by barks, and wood from the stem, whereas plant parts such as seeds, fruits, and resins were rarely used. Trees were found to be the most frequent source of traditional cosmetics in the study area followed by shrubs (in life forms (Fig. 2; Table 4).

**Table 4:** Plant species, voucher number (V. No.), family, parts used (PU), methods of preparation (MP), use category (UC), and application area (AP) for traditional cosmetics by Oromo women in Madda Walabu district, Southeastern Ethiopia

Species	V. No	Family	PU	MP	Administration	UC	AP
*Acacia brevispica* Harms	S027	Fabaceae	Leaves	Decoction	Applied topically via sprinkling on wounds and pimples	Perfume	Skin
*Acacia drepanolobium* Harms ex Sjöstedt	S016	Fabaceae	Fruit	Maceration	Oil is applied topically to repair and beautify the skin	Skincare	Skin
*Acacia etbaica* Schweinf	S036	Fabaceae	Wood	Directly used	The trunk for brushing teeth	Teeth Hygiene	Teeth
*Acacia mellifera* (Vahl) Benth	S048	Fabaceae	Flower	Maceration	The oil applied topically as hair cream	Hair coloring	Hair
*Aloe citrina* Carter & Brandham	S040	Aloaceae	Whole	squeezing	The fluid squeezed is used for skin beauty	Skincare	Skin
*Azadirachta indica* A. Juss	S028	Meliaceae	Leaves	Maceration	Boiling the fresh leaves	Face cleaner	Face
*Boswellia neglecta* S. Moore	S015	Burseraceae	Resin	Smoked	Resins are dropped on fire	Perfume	Skin
*Cadaba longifolia* (R. Br.) DC	S043	Capparidaceae	Bark	Smoked	Pieces of bark are burned on fire	Perfume	Skin
*Carica papaya* L	S008	Caricaceae	Leaves	Decoction	The bark is applied topically on burn wounds (burns)	Skincare	Skin
*Citrus aurantifolia* (Christm.) Swingle	S007	Rutaceae	Bark	Maceration	The bark is applied topically on burn wounds (burns)	Face cleaner	Face
*Combretum adenogonium* Steud. ex A. Rich	S014	Combretaceae	Leaves	Decoction	The leaves are applied topically on wounds and sores	Face cleaner	Face
*Commiphora baluensis* Engl	S029	Burseraceae	Bark	Maceration	The bark is used for wounds and it rejuvenates the skin and is applied topically on the skin or wounds	Face mask	Face
*Commiphora habessinica* (Berg) Engl	S005	Burseraceae	Leaves	Maceration	Leaves are applied topically to stimulate hair growth	Hair health	Hair
*Commiphora myrrha* (Nees) Engl	S037	Burseraceae	Bark	Smoked	The bark is burned and the vagina is fumigated	Vaginal health	Vagina
*Cordia africana* Lam	S010	Boraginaceae	Bark	Maceration	Barks are grinded and mixed with water	Hand decorative	Hand
*Cordia monoica* Roxb	S041	Boraginaceae	Leaves	Decoction a	The leaves are used to wash the vagina	Vaginal health	Vagina
*Croton dichogamus* Pax	S017	Euphorbiaceae	Bark	Maceration	The oil from the bark applied topically to repair and beautify the skin	Face mask	Face
*Croton macrostachyus* Del	S038	Euphorbiaceae	Seeds	Maceration	Stimulate hair growth; it is applied topically	Hair cream	Hair
*Dalbergia commiphoroides* Bak. f	S009	Fabaceae	Leaves	Decoction	The bark is grinded and used	Skincare	Skin
*Delonix elata* (L.) Gamble	S042	Fabaceae	Leaves	Decoction	The leaf is crushed and rubbed on the skin	Skincare	Skin
*Dobera glabra* (Forssk.) Poir	S018	Salvadoraceae	Roots	Maceration	Roots are applied topically and orally for mouth sores and as toothpaste	Teeth Hygiene	Teeth
*Dodonaea angustifolia* L. f	S026	Sapindaceae	Wood	Directly used	The trunk is for brushing	Teeth Hygiene	Teeth
*Ehretia cymosa* Thonn	S039	Boraginaceae	Leaves	Maceration	Leaves are applied topically on wounds	Hand decorative	Hand
*Euphorbia tirucalli* L	S046	Euphorbiaceae	Leaves	Directly used	Leaves are applied orally and as lotion on burned skin and wounds	Perfume	Skin
*Gnidia stenophylla* Gilg	S025	Thymelaeaceae	Leaves	Maceration	The leaves are burned and applied topically on the wound	Skincare	Skin
*Grewia bicolor* Juss	S030	Tiliaceae	Bark	Maceration	Grinded and mixed with water	Face mask	Face
*Juniperus procera* Hochst. ex Endl	S019	Cupressaceae	Wood	Smoked	By frightening the trunk	Perfume	Skin
*Kalanchoe lanceolata* Forssk.) Pers	S006	Crassulaceae	Whole plant	Decoction	Grinding of root and bark	Skincare	Skin
*Kirkia burgeri* Stannard	S035	Simaroubaceae	Roots	Maceration	The oil from the root applied topically as hair cream	Skincare	Skin
*Mimusops kummel* A. DC	S031	Sapotaceae	Leaves	Decoction	The sap squeezed directly on skin wounds	Skincare	Skin
*Murdannia simplex* (Vahl) Brenan	S024	Commelinaceae	Roots	Smoked	It is taken orally as a mouthwash	Teeth Hygiene	Teeth
*Olea europaea* L*.* subsp. *cuspidata* (Wall. ex G.Don)	S021	Oleaceae	Seeds	Maceration	Stimulate hair growth and applied topically	Hair cream	Hair
*Osyris quadripartita* Decn	S044	Santalaceae	Bark	Maceration	Bark is taken orally because it is believed the skin is affected from the inside	Hair health	Hair
*Persea americana* Mill	S011	Lauraceae	Leaves	Directly used	Leaves are applied topically on wounds	Face mask	Face
*Premna schimperi* Engl	S047	Lamiaceae	Leaves	Decoction	Put the medicine on the infected teeth	Teeth Hygiene	Teeth
*Rhamnus staddo* A. Rich	S032	Rhamnaceae	Leaves	Decoction	The leaves are crushed and applied	Perfume	Skin
*Rhus natalensis* Krauss	S020	Anacardiaceae	Wood	Directly used	A piece of the trunk is cut and used	Teeth Hygiene	Teeth
*Sesamothamnus rivae* Engl	S001	Pedaliaceae	Bark	smoked	Powder from the bark is applied as a paste on the mouth sores	Skincare	Skin
*Solanum hastifolium* Hochst. ex Dunal in DC	S003	Solanaceae	Roots	Decoction	The cream is applied on the skin (acne)	Skincare	Skin
*Solanum lycopersicum* L	S045	Solanaceae	Fruit	Squeezed	Fruit sap is administered topically as a facial wash	Face cleaner	Face
*Stereospermum kunthianum* Cham	S034	Bignoniaceae	Bark	Directly used	A piece of bark is cut and used	Teeth Hygiene	Teeth
*Strychnos mitis* S. Moore	S023	Loganiaceae	Wood	Smoked	A piece of wood is burned on a small fire	Skincare	Skin
*Syzygium guineense* (Willd.) DC	S012	Myrtaceae	Bark	Smoked	A piece of bark is burned on the small fire	Perfume	Skin
*Terminalia brownii* Fresen	S002	Combretaceae	Seeds	Maceration	Mixed with soil for cleaning teeth and orally as toothpaste	Teeth Hygiene	Teeth
*Terminalia laxiflora* Engl. & Diels	S004	Combretaceae	Bark	Maceration	It is applied topically on wounds	Skincare	Skin
*Withania somnifera* L. Dunal in DC	S013	Solanaceae	Wood	Decoction	Leaves crushed and extracts applied on skin	Perfume	Skin
*Ximenia americana* L	S022	Olacaceae	Wood	Directly used	The trunk for brushing teeth	Teeth Hygiene	Teeth
*Zanthoxylum chalybeum* Engl	S033	Rutaceae	Bark	Squeezed	squeezed cock and applied the fluid	Hair coloring	Hair

#### Methods of preparation and administration

**Fig. 3:**
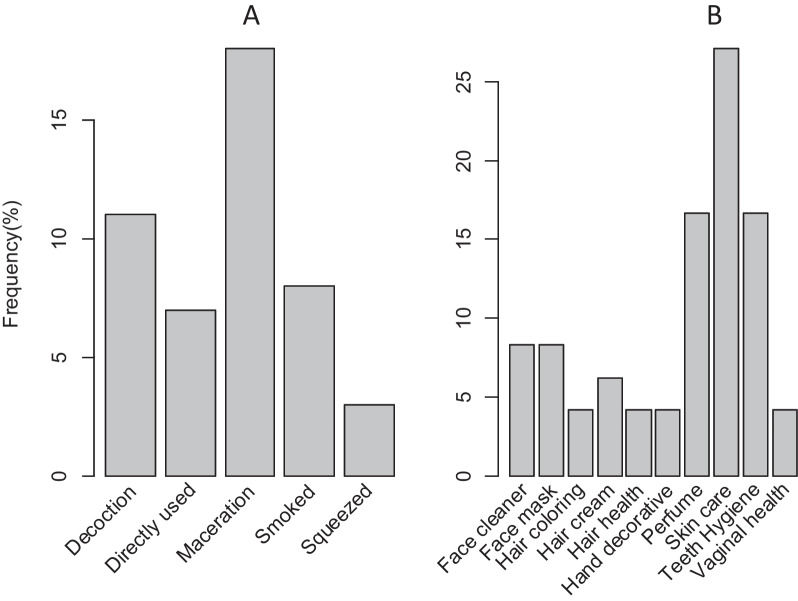
Methods of preparation **A** and major use categories **B** of the plant-based traditional cosmetics products among Oromo women in Madda Walabu District of Bale Zone

Maceration and decoction were the most common methods used to prepare plant-based traditional cosmetics (Fig. 3A; Table 4). These methods were usually used by the Oromo women to soften and extract materials that can be used in the production and beautification of the skin, and hair. Furthermore, smoking was also mentioned as a common method, particularly in the use of traditional cosmetics such as perfume. Traditional cosmetics are applied to different parts of the body. Moreover, these natural-based cosmetics are mainly administered topically (88%) while oral infusions were not common (12%) (Table 4). The participant highlighted that plants used for natural-based cosmetics may be administered as a powder (leaves, root, or wood powder) through different mechanisms depending on the need for beautification.

### Cosmetic use category and application areas

**Fig. 4:**
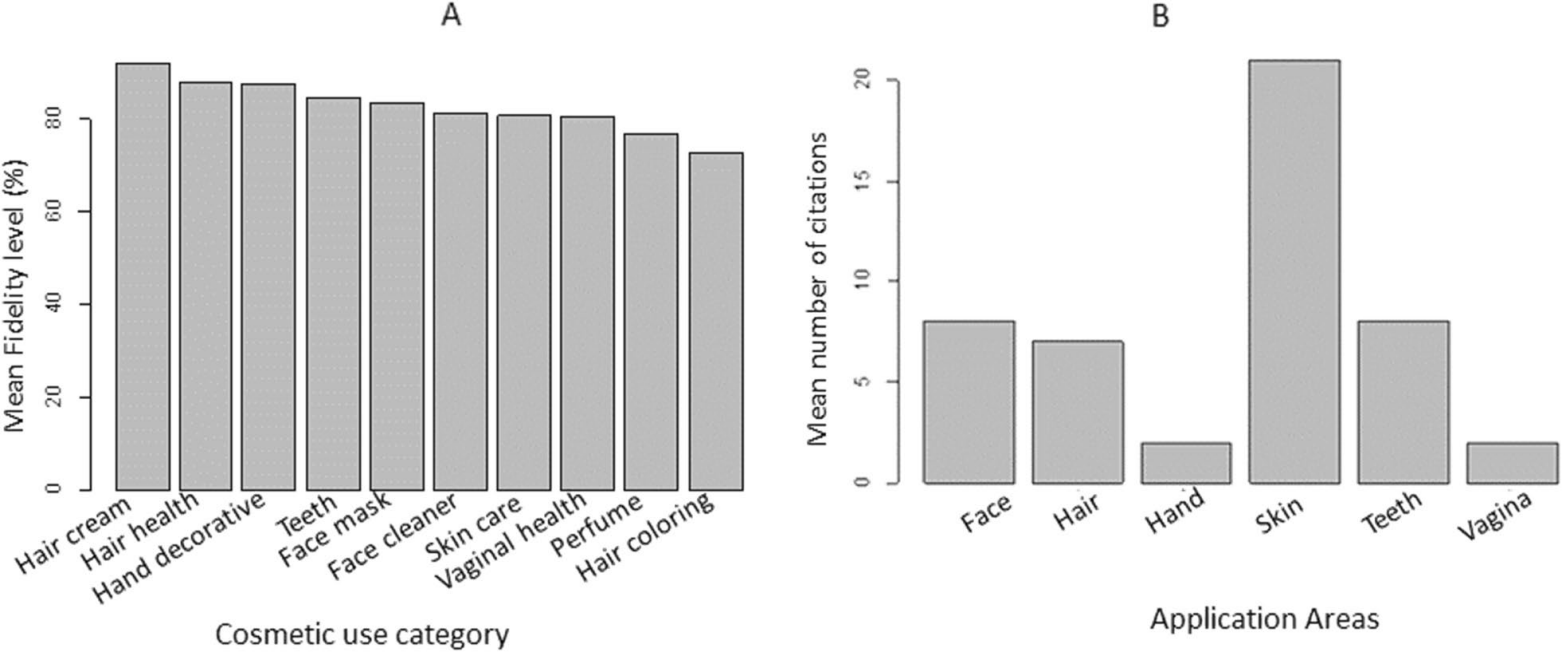
Major cosmetic use categories **A** and the main cosmetic body application areas **B** among the Oromo women in the study area

Most of the categories (i.e., three fourth) had high mean fidelity levels for particular use values, ranging from 72.73 to 92, 03% (Fig. 4A). The highest mean FL value was recorded for use as hair cream, followed by hair health and hand decorative; and skin and face mask were the 4th and 5th, respectively (Table 5). The traditional cosmetics among the Oromo women in the Madda Walabu district were used for care and beautifying skin followed by teeth hygiene, face cleaning, and masks (Fig. 4B).

**Table 5:** The main cosmetic plants cited by most of the informants in the study area (*GF* growth form, *No.C* number of citations and *No.C*(%) percentages of number of citations)

Species	GF	Local name	Application area	No. C	No. C (%)
*Terminalia brownii* Fresen	T	Birdheessa	Hair	47	3.77
*Aloe citrina* Carter & Brandham	H	Hargiisa	Skin	45	3.61
*Olea europaea* L. subsp*. cuspidata* (Wall. ex G.Don)	T	Ejersa	Hair	45	3.61
*Sesamothamnus rivae* Engl	T	Dareessa	Skin	45	3.61
*Terminalia laxiflora* Engl. & Diels	S	Dabaqqaa	Skin	45	3.61
*Syzygium guineense* (Willd.) DC	T	Baddeessaa	Skin	43	3.45
*Rhus natalensis* Krauss	T	Daboobessa	Teeth	42	3.37
*Combretum adenogonium* Steud. ex A. Rich	T		Face	41	3.29
Sum					28.35

### Cosmetic applications of plants in the study area

Eight plant species were cited by one-third of the informants as main cosmetic plants (Table 5). *Terminalia brownii* Fresen*. *was the most cited traditional cosmetic plant used as hair cream followed by *Aloe citrine*, used for skin care, and *Olea europaea* L*.* subsp*. cuspidata* (Wall. ex G. Don) (Table 5). However, when taking each category of usage into account, the most cited plants were different. For example, *Terminalia laxiflora* Engl. & Diels. were the most cited in the skincare category, *Rhus natalensis* Krauss in the keeping teeth hygiene, *Combretum adenogonium* Steud. ex A. Rich. in the face clearance, *Cordia monoica* Roxb. in the vaginal health, and *Ehretia cymosa* Thonn. in the category of hand decorative (Table 5; Appendix Table 11).

### The fidelity level of a species for a particular use

In the study, greater cosmetic usage citations were exhibited in the fidelity level (FL) revealing the importance of a species for a particular use. Nineteen fidelity level values higher than 88% related to use-species associations known by more than half of the informants (Table 6; Appendix Table 11). Thus 19 species and nine cosmetic uses appeared to be particularly linked. Fidelity levels were high (> 80% of FL) for several species (Appendix Table 11). The highest FL score was recorded for *Terminalia brownii* Fresen. used for the beautification of hair followed by *Terminalia laxiflora* Engl. & Diels. for skin care*, Sesamothamnus rivae* Engl. for skin care*, Olea europaea* L. subsp*. cuspidata* (Wall. ex G. Don) for hair cream, and *Aloe citrina* Carter & Brandham for skin care (Table 6; Appendix Table 11).

**Table 6:** Plants with high fidelity level values (> 88%) against cosmetic use category of Oromo women in Madda Walabu district, Southeastern Ethiopia (*FL* fidelity level, *Ip* number of informants who independently cited the importance of a species for treating a particular use category, *Iu* total number of informants who reported the plant for any given use category)

No	Scientific name	Use category	Ip	Iu	FL (%)
1	*Terminalia brownii* Fresen	Hair cream	23	24	95.83
2	*Terminalia laxiflora* Engl. & Diels	Skincare	22	23	95.65
3	*Sesamothamnus rivae* Engl	Skincare	22	23	95.65
4	*Olea europaea* L. subsp. *cuspidata* (Wall. ex G.Don)	Hair cream	22	23	95.65
5	*Aloe citrina* Carter & Brandham	Skincare	22	23	95.65
6	*Syzygium guineense* (Willd.) DC	Perfume	21	22	95.45
7	*Combretum adenogonium* Steud. ex A. Rich	Face cleaner	20	21	95.24
8	*Euphorbia tirucalli* L	Perfume	19	20	95.00
9	*Commiphora habessinica* (Nees) Engl	Hair health	16	17	94.12
10	*Premna schimperi* Engl	Teeth	16	17	94.12
11	*Murdannia simplex* (Vahl) Brenan	Teeth Hygiene	15	16	93.75
12	*Gnidia stenophylla* Gilg	Skincare	15	16	93.75
13	*Commiphora baluensis* Engl	Face mask	13	14	92.86
14	*Rhus natalensis* Krauss	Teeth Hygiene	20	22	90.91
15	*Cordia africana* Lam	Hand decorative	8	9	88.89
16	*Stereospermum kunthianum* Cham	Teeth Hygiene	16	18	88.89
17	*Azadirachta indica* A. Juss	Face cleaner	8	9	88.89
18	*Cordia monoica* Roxb	Vaginal health	15	17	88.24
19	*Dodonaea angustifolia* L. f	Teeth Hygiene	15	17	88.24

### Informant consensus factor (ICF)

To compute the informant consensus factor values, cosmetic use categories of the study area were grouped into 10 categories (Table 7; Fig. 4A). All categories had high informant consensus factor values, ranging from 0.92 to 0.96. Specifically, face clearance and mask scored the highest ICF value, followed by hair treatment (Table 7). The traditional cosmetics among the Oromo women in Madda Walabu District were thus used to care for and beautify the face, followed by hair and skin (Table 7).

**Table 7:** Cosmetic use category and informant consensus factor of Oromo women in Madda Walabu district, Southeastern Ethiopia

Use category	Number of use reports in each category (Nur)	Number of taxa (Nt)	Informants’ consensus factor (IFC)
Face cleaner	56	3	0.96
Face mask	26	2	0.96
Hair coloring	25	2	0.96
Hair cream	23	2	0.95
Hair health	109	8	0.94
Hand decorative	16	2	0.93
Perfume	171	13	0.93
Skincare	43	4	0.93
Teeth Hygiene	38	4	0.92
Vaginal health	86	8	0.92

### Relative frequency of citations and cultural importance index

The most frequently cited being used as traditional cosmetics and considered more culturally important by the Oromo women in the study area was *Terminalia brownii* followed by *Aloe* citrine, *Terminalia laxiflora* (Table 8; Appendix Table 12).

**Table 8:** Relative frequency of citation (RFC) and cultural important index (CII) of the most relevant nine plant species in traditional cosmetics use among the Oromo women in the Madda Walabu district, Southeastern Ethiopia

Plant species	RFC	CII
*Terminalia brownii*	0.15	0.31
*Aloe citrina*	0.15	0.30
*Terminalia laxiflora*	0.15	0.30
*Sesamothamnus rivae*	0.15	0.30
*Olea europaea* subsp*. cuspidata*	0.15	0.30
*Syzygium guineense*	0.14	0.29
*Combretum adenogonium*	0.13	0.28
*Rhus natalensis*	0.13	0.27
*Euphorbia tirucalli*	0.13	0.26

### Sociodemographic factors in the use of traditional cosmetics among Oromo women

**Fig. 5:**
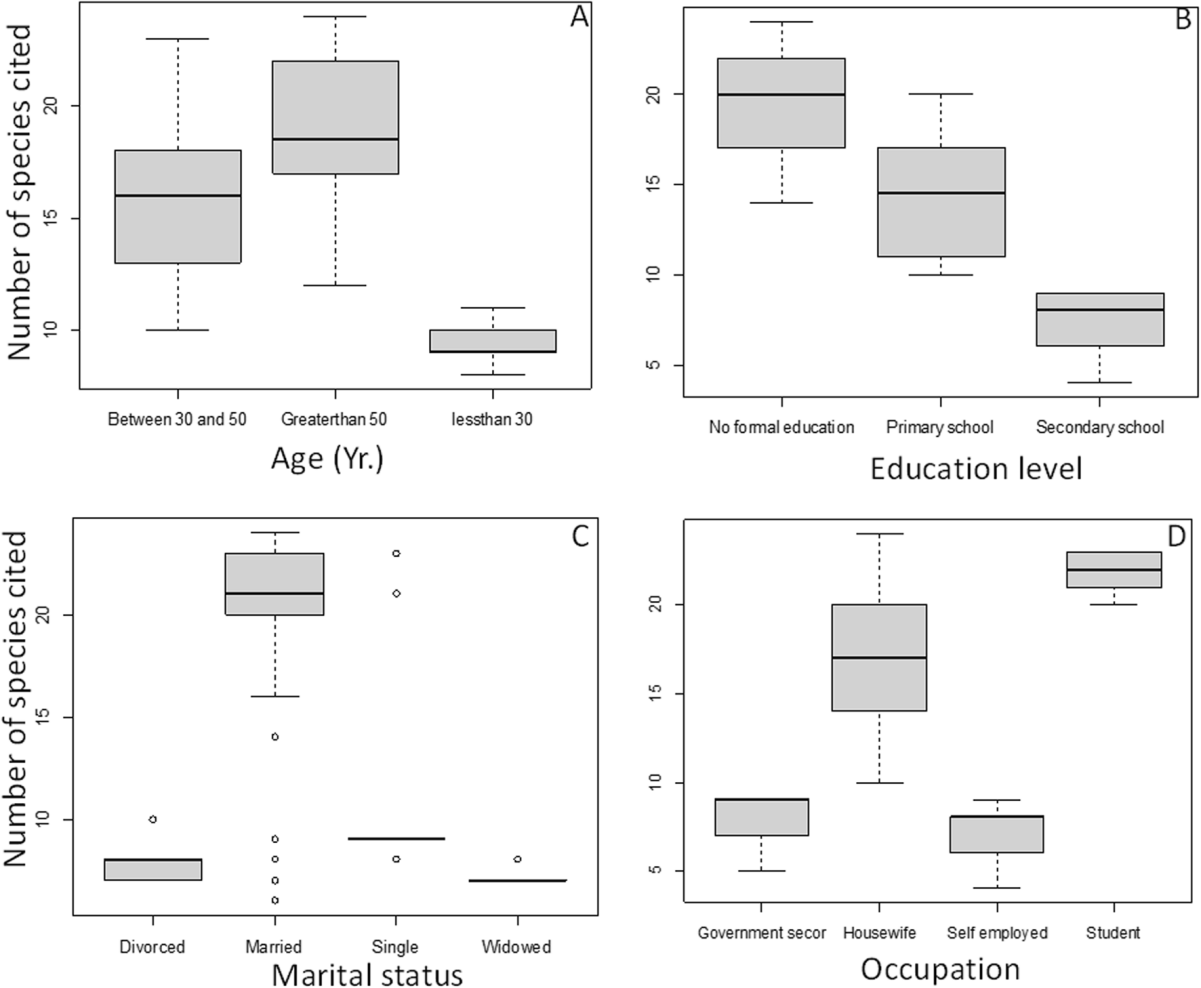
Variation in the use of traditional cosmetics based on **A** age, **B** level of education, **C** marital status, and **D** occupation among the Oromo women in Madda Walabu District, Bale Zone, Southeastern Ethiopia

There were highly significant differences in the number of plant species used as traditional cosmetics among the Oromo women with different ages (*F* =  70.6 *p* < 0.001), level of education, (*F* = , 154.0 *p* < 0.001), marital status (*F* = 68.4 *p* < 0.001), and occupation (*F* = , 45.4 *p* < 0.001). Older women mentioned more plant species used as traditional cosmetics than younger ones. Women who did not attend formal education were found to be retaining indigenous plant use knowledge more than educated ones. Moreover, married and housewives were found to have extensive traditional knowledge compared to other married status-employed women in the study area (Fig. 5).

### Traditional knowledge and cultural practices

The respondents made clear that traditional knowledge about natural-based cosmetics is widely spread among the Oromo women in the Madda Walabu district although they do not regularly exchange knowledge with other communities around. The Oromo women played a primary role in the preparation and administration of natural-based cosmetics. Their homemade remedies were prepared for the whole community who require different forms of preparations such as poultices or compresses. Some plant species that are used for natural-based cosmetics among the Oromo women are also used as an aspect of healing. Hence, the Oromo women who were knowledgeable articulated that natural-based cosmetics are an interdisciplinary practice that heals and promotes the beauty of the skin. Respondents mentioned that plant species are collected throughout the year, and seasonality only plays a role when plant parts such as fruits or leaves are to be collected. It was observed that the Oromo women who were knowledgeable about natural-based cosmetics harvested and carried as many plants as they could when they went out for collection. The decision to have the large collection at once is due to the long distance they had to travel for the collection and the increasing scarcity of certain plant species. Moreover, the respondents mentioned that large collections of scarce species are recently threatening the species. Despite the presence of gender divisions for different activities because of cultural restrictions, the majority of women respondents mentioned that there were no taboos associated with nature-based cosmetic plant collection and uses in the study area.

### Description of particular care types among Oromo women

Intimate hygiene is typical of the Oromo women in Oromia which is called *qayyachuu* in Afan Oromo. Care of private parts through fumigation is very important for women, particularly for married women. These treatments are preventive actions to avoid bad smells, limit vaginal discharges, and can arouse the woman; making her ready for sexual activities. For the fumigation process, a small hole is made inside the home where wood material is placed on a fire and women cover their bodies with a cloth below their necks. The wood materials for *qayyachuu* are mainly prepared from the mix of the barks and branches from plant species such as *Commiphora baluensis* Engl. *C. habessinica* (Berg) Engl. and *C. myrrha* (Nees) Engl. These intimate care treatments are only for the female gender.

## Discussions

### Composition of plants used in traditional cosmetics in Madda Walabu district

Despite the present study describing and documenting plants used as traditional cosmetics, these plants can be used for both cosmetic and medicinal purposes, and there can be some overlaps between these two categories in many cultures [34–36]. However, there are some key distinctions between the two: cosmetic applications are mainly used to enhance physical appearance or hygiene and may have some minor therapeutic effects, but are not intended primarily for treating medical conditions. Examples: moisturizers, hair conditioners, soaps, and perfumes [37]. Thus, the study mainly focused on the cosmetic role of the plants even though these plants can have medicinal applications in addition. The primary purpose of the plant applications was determined based on the information provided by the informants. Thus, based on the informants’ response, if the plant was primarily used to enhance physical appearance or hygiene, it was classified as a cosmetic application.

In various parts of the world including Ethiopia, many plant species are commonly used locally for the preparations of traditional cosmetics but have not been scientifically investigated for wider use [38]. This implies a need to collect and compile indigenous knowledge of plant-based traditional cosmetics among ethnic groups, specifically, the Oromo (largest ethnic group in Ethiopia) women of Bale lowland in this study. Accordingly, a total of 48 plant species used as traditional cosmetics were recorded of which Fabaceae, Bigonaceae, and Burseraceae were the most frequent families. The study thus revealed that there is a great diversity of plants for cosmetic use, which could be useful documentation, contributing to preserving the knowledge about the traditional cosmetic use of plants in this region. Consequently, the ethnobotanical study of these species reveals that the Oromo women in the Madda Walabu district possess a deep knowledge of local plants and their cosmetic applications. The traditional cosmetics they use reflect their cultural heritage and their connection to the natural environment. The study contributes to the documentation of ethnobotanical knowledge and provides insights into the potential for developing sustainable cosmetic products based on traditional practices.

The study documented a relatively higher number of species specifically used as traditional cosmetics compared to other similar studies (Table 9). This high number of species could be attributed to the varied agroecology and climatic conditions of our study area that supported a variety of plant species. A comparison of the species number discovered in different countries is presented below.

**Table 9:** Number of plant species used as a traditional cosmetic reported in different countries

Number of species	Country	References
27	Alexandria, Egypt	[40]
13	Arab-Choa and Kotoko Ethnic Groups in the Semi-Arid Areas of Far North Cameroon	[41]
47	Vhavenda women in Vhembe District Municipality, Limpopo, South Africa	[39]
40	Province of Taza, Northern Morocco	[36]
25	Tropical island of Mauritius	[42]
16	Xhosa women in the Eastern Cape, South Africa	[43]
29	Tribal women of Kashmir Himalayas	[2]

Moreover, the top rank of Fabaceae which aligns with other studies [39], as a source of local cosmetics is not surprising for several reasons. First, Fabaceae is among the most species-rich families (3rd) in the flora area of the country [44, 45]. The same is true for the Burseraceae family since the vegetation of the study area is mainly *Acacia commiphora* woodlands [21]. The potential importance of the species as cosmetics in this family could therefore be a result of its representation in the flora of the area. Second, in neighboring districts with similar agroecology such as Dallo Manna [46] and Gura Damole districts [47], Fabaceae was found to contain many medicinal plants. However, the strange thing was that Lamiaceae which contains many cosmetic and medicinal plants that are of global importance [40, 48] was only represented by one species in the study area. The variation could be attributed to the differences in agroecology. Moreover, similar to other studies in Africa (tropical) that focused on traditional cosmetics [39–41], trees were the most commonly used plant life forms followed by shrubs and herbs. The high usage of such plant life forms in Madda Walabu is also likely associated with the tropical climate region which, in turn, helps the plants to be widely available and abundant in the study area.

### Plant parts used and use category

Among a range of plant organs used in the study area, leaves are the parts that are most commonly used in traditional cosmetics which are in line with other studies [39, 40, 46, 47]. The preference for leaves as a major source of cosmetics could be asserted by the fact that they are not only easy to collect, store, and process during most of the year but also are the site of photosynthesis and sometimes the storage of bioactive ingredients responsible for the cosmetic properties of the plant helping beautification [49, 50]. The use of leaves as traditional cosmetics encourages conservation practices, unlike the extensive use of roots and barks which may cause the death of plants. However, reports from some other studies revealed that fruits were the most commonly used plant parts as cosmetics among women [2, 51]. The difference could be attributed to the variations in indigenous knowledge on plant-based cosmetics differing across different communities and ethnic groups globally.

A greater number of citations for cosmetic applications are exhibited in the fidelity level. In the current study, about 19 species were found to have FL values of greater than 88% suggesting that the state of knowledge of the informants is more or less common when it comes to the uses of such plants. However, species such as *Terminalia brownii* Fresen and *Olea europaea* L. subsp*. cuspidata* (Wall. ex G. Don for hair treatment and *Terminalia laxiflora* Engl. & Diels and *Sesamothamnus rivae* Engl. for skincare were the most frequently cited species among the Oromo women in the study area. Moreover, from the computation of relative frequency citations and cultural importance index, these same species were found to be the most frequently cited for use as traditional cosmetics and considered culturally more important among the Oromo women in the district. This could be due to their availability. For example, the genus *Terminalia* is the second largest genus of Combretaceae and is widely distributed in Eastern African countries such as Ethiopia [52]. Propagation can occur naturally through seeds or vegetative methods using wild plants, seedlings, tree stumps, or young plants [53]. *Terminalia* spp. provides economic, medical, spiritual, and social benefits in Ethiopia [54]. Similarly, *Aloes* are recognized as an important component of the dry-land ecosystems, primary colonizers of habitats that might enable later habitation by other less resilient plants. Areas that experience prolonged drought can benefit from the planting of *Aloe* [55]. *Aloes* are used in soap production, jute sack production, and hair washing [56]. The other reason could be related to their use, for skincare in which the skin is the largest organ exposed to external barrier, requires frequent care and treatment [40, 41, 57].

FL is a measure of the degree of effectiveness of the cosmetic plant for beautification. Thus, traditional cosmetic plants having high fidelity level values are speculated to be effective in their beautification potential and can be a good candidate for further detailed investigation. Although [17] reported the FL values of some of the aforementioned species for their medicinal aspect, no literature has documented their FL scores for cosmetic uses as the present study is the first of its kind in the area and also due to variations of the use of plants from area to area. From informant consensus factor analysis, high FIC values suggested that Oromo women in the community share knowledge about the most significant plant species of traditional cosmetics commonly used for beautification and low FIC values indicate less willingness to share knowledge of significant plant species of traditional cosmetics for beautification for women [58]. From the current study, although all the usage categories had higher values, face care has the highest FIC which further substantiates the heavy reliance of Oromo women of Madda Walabu district on plant-based traditional cosmetics.

### Method of preparations and mode of application

As documented in other ethnobotanical studies in Bale [59], diverse methods of preparation were observed in the current study. Accordingly, maceration and decoction were the most frequently used preparation methods among the participants. This goes in line with other similar studies where decocting or concocting was mostly the more common way of preparation [50]. Furthermore, in the current study, most of the products of traditional cosmetics were administered topically which aligns with other several studies [57, 60]. This route of administration was demonstrated with eleven different cosmetic applications. Remarkably, this proves the certainty that plant species are intensely entrenched in the cosmetic globe with different kinds of cosmetic uses. Applications of natural-based cosmetics such as a paste, powder, or sap (topically) were also in alignment with the findings [34].

### Plant-based traditional cosmetics and Oromo women's indigenous knowledge

The current study revealed significant variations in the number of plant species used as traditional cosmetics among Oromo women of different ages. Accordingly, older women mentioned and used more plant species as traditional cosmetics for beautification than younger ones. This aligns with the findings of [2] and [40] indicating that indigenous knowledge of plant-based traditional cosmetics is not equally distributed among the different age groups of women. Thus, the study revealed a well-established indigenous knowledge of plant-based cosmetics among the Oromo women but seems to decline with age which could be attributed to the low interest of the younger generation to inherit and use traditional cosmetics. The other finding of the current study is that Oromo women, who didn't attend formal education, are married, and housewives have more extensive knowledge of traditional cosmetics than students, educated and employed. This could be due to the inspiration of the younger women, educated and employed ones by intensive media campaigns and advertisements of synthetic cosmetics that might have resulted in decreasing interest in using natural cosmetics [2]. Thus, due to the declining trends of the use of plant-based traditional cosmetics in the younger generation, which was also supported by other studies [61], there is an urgent need to preserve the indigenous knowledge of the Oromo women of Madda Walabu district. We suggest that the younger generation should be trained and made aware of the importance, of sustainable utilization as well as the domestication of precious plant-based traditional cosmetics.

The type of occupation also plays a major role in the use of herbal cosmetics. We discovered that housewives use more cosmetic plants for their skin, face, hair, and teeth hygiene than women who work outside of the home. Housewives tend to have less income than working women, hence their higher interest in cheaper cosmetics. Similar studies in Egypt reported that herbal remedies are much more common among lower-income groups, such as students, housewives, and non-literate women [40]. On the contrary, the results of the study that took place in Finland revealed that women who had relatively high social status were the most interested in herbal remedies and cosmetics [62], suggesting that knowledge and use of herbal remedies may be context-specific. On the other hand, [63] reported that knowledge of medicinal plants was not related to age or gender in Brazil.

Besides the regular application of plant-based natural cosmetics, Oromo women knowledgeable about traditional cosmetics indicated that the different types of plant species are inspired mainly by their culture, and cosmetic products are mainly used in cultural practices such as traditional healing and skin-related issues. In contrast to other studies [58] that restrict outdoor activities including plant gathering from wild to men, our study revealed that nature-based cosmetic plant collection was carried out by women which aligns with [39].

Concerning traditional knowledge and cultural practices, the Oromo women made clear that their homemade cosmetics were prepared for the whole community and required different forms of preparation. Moreover, the knowledgeable respondents articulated that natural-based cosmetics are interdisciplinary practices that heal and promote the beauty of skin which is in line with [17].

Furthermore, the current study revealed that cosmetics play a significant role in cultural identity and expression. The use of specific plants for cosmetic purposes is often associated with cultural norms, values, and beliefs. For Oromo women, the use of traditional cosmetics is a way to express their cultural identity and connect with their heritage. For example, there was particular care for intimate hygiene which is typical of the Oromo women in the area that is called *qayyachuu* in Afan Oromo. It is the means of caring for private parts (vagina) through smoking and fumigation, particularly for married women which align with [17] but with a different name, *woyeba chis* meaning smoking bath in northern Ethiopia. The treatments are used to avoid bad smells, limit vaginal discharges, and can arouse the woman, making her ready for sexual activities. The wood materials for *qayyachuu* are mainly prepared from the mix of the barks and branches from plant species such as *Commiphora baluensis* Engl. *C. habessinica* (Berg) Engl. and *C. myrrha *(Nees) Engl.

## Conclusions

The study described the cosmetic flora of the Madda Walabu district of Bale Zone with its uses. The ethnobotanical study focusing on traditional cosmetics is the first of its kind to be conducted among Oromo women in Ethiopia. A total of 48 plants belonging to 31 families used in traditional cosmetics were investigated, and eight plant species were cited by one-third of the informants as the main cosmetic plants. The traditional cosmetics among the Oromo women in the Madda Walabu district were thus used to care for and beautify the face, followed by hair and skin. The most frequent part of the plant used for the preparation of traditional cosmetics was the leaves followed by barks, and wood from the stem. The preparation for the cosmetics was through maceration and decoction which were used to soften and extract the production used as traditional cosmetics. Furthermore, smoking was also mentioned as a common method, particularly in the use of traditional cosmetics such as perfume.

Findings from this study indicated the rich plant biodiversity in terms of the high number of plants used for traditional cosmetics among the Oromo women. Furthermore, women's information regarding the plant used as a traditional cosmetic was highly credible. Traditional cosmetics play a significant role in cultural identity and expression. The use of specific plants for cosmetic purposes is often associated with cultural norms, values, and beliefs. For Oromo women, the use of traditional cosmetics is a way to express their cultural identity and connect with their heritage. Additionally, traditional cosmetic practices can empower women by providing them with a sense of agency and control over their appearance. The use of natural ingredients and the knowledge of how to prepare and apply cosmetics give women a sense of pride and confidence. However, the cultural heritage associated with traditional cosmetics is facing threats from various factors, including: the influx of modern cosmetics and beauty products inspired by intensive media campaigns and advertisements. Thus, educating younger generations about the importance of traditional cosmetics and their role in cultural heritage, and supporting community-led initiatives to revitalize traditional cosmetic practices and promote their cultural significance is crucial. Moreover, the over-exploitation of plant resources for cosmetic purposes can lead to environmental degradation and the loss of biodiversity. Thus, promoting sustainable harvesting practices to ensure the availability of plant resources for future generations should not be ignored. The continuation and preservation of this traditional knowledge ensure the preservation of valuable cultural heritage and promote the potential for sustainable development in the region.

The study mainly focused on the cosmetic application of the plants even though these plants can have medicinal values. Therefore, we recommend amplified research in future and assessing multipurpose ethnobotanical use values since our study is an eye opener for the study area and aimed to mainly investigate cosmetical aspects.

## Data Availability

Data available on request from the authors.

## Appendix 1

See Tables

**Table 10:** List of plants used as traditional cosmetics among Oromo women in Madda Walabu District of Bale Zone, Southeast Ethiopia, where *GF* growth form, *T* tree, *S* shrub, and *H* herb

Scientific name	Family	GF	Afan Oromo name
*Acacia brevispica* Harms	Fabaceae	S	Hammarreessaa
*Acacia drepanolobium* Harms ex Sjöstedt	Fabaceae	S	Fuullessa
*Acacia etbaica* Schweinf	Fabaceae	T	Burquqqee
*Acacia mellifera* (Vahl) Benth	Fabaceae	T	Hilaala
*Aloe citrina* Carter & Brandham	Aloaceae	H	Hargiisa
*Azadirachta indica* A. Juss	Meliaceae	T	Niimii
*Boswellia neglecta* S.Moore	Burseraceae	T	Qumbii
*Cadaba longifolia* (R. Br.) DC	Capparidaceae	S	Maramaa
*Carica papaya* L	Caricaceae	T	Paappayaa
*Citrus aurantifolia* (Christm.) Swingle	Rutaceae	T	Loomii
*Combretum adenogonium* Steud. ex A. Rich	Combretaceae	T	Dareessa
*Commiphora baluensis* Engl	Burseraceae	T	Hagarsuu
*Commiphora habessinica* (Berg) Engl	Burseraceae	T	Xillaaa
*Commiphora myrrha* (Nees) Engl	Burseraceae	T	Qumbii
*Cordia africana* Lam	Boraginaceae	T	Waddeessa
*Cordia monoica* Roxb	Boraginaceae	T	Leedii
*Croton dichogamus* Pax	Euphorbiaceae	S	Maakaftaa
*Croton macrostachyus* Del	Euphorbiaceae	T	Makkanniisa
*Dalbergia commiphoroides* Bak. f	Fabaceae	S	Calcala
*Delonix elata* (L.) Gamble	Fabaceae	T	Shukeellaa
*Dobera glabra* (Forssk.) Poir	Salvadoraceae	T	Aadee
*Dodonaea angustifolia* L. f	Sapindaceae	S	Ittacha
*Ehretia cymosa* Thonn	Boraginaceae	T	Ulaagaa
*Euphorbia tirucalli* L	Euphorbiaceae	S	Aannoo
*Gnidia stenophylla* Gilg	Thymelaeaceae	H	Qaxaricha
*Grewia bicolor* Juss	Tiliaceae	S	Harooressa
*Juniperus procera* Hochst. ex Endl	Cupressaceae	T	Hindheessa
*Kalanchoe lanceolata* (Forssk.) Pers	Crassulaceae	H	Boobiyyaa
*Kirkia burgeri* Stannard	Simaroubaceae	T	Musdhugaa
*Mimusops kummel* A. DC	Sapotaceae	T	Qolaatii
*Murdannia simplex* (Vahl) Brenan	Commelinaceae	H	Qayyoo
*Olea europaea* L. subsp*. cuspidata* (Wall. ex G. Don)	Oleaceae	T	Ejersas
*Osyris quadripartita* Decn	Santalaceae	T	Waatoo
*Persea americana* Mill	Lauraceae	T	Avukaadoo
*Premna schimperi* Engl	Lamiaceae	S	Urgeessaa
*Rhamnus staddo* A. Rich	Rhamnaceae	T	Qadiidaa
*Rhus natalensis* Krauss	Anacardiaceae	T	Daboobessa
*Sesamothamnus rivae* Engl	Pedaliaceae	T	Dareessa
*Solanum hastifolium* Hochst. ex Dunal in DC	Solanaceae	H	Hidii budaa
*Solanum lycopersicum* L	Solanaceae	H	Timaatima
*Stereospermum kunthianum* Cham	Bignoniaceae	T	Botoroo
*Strychnos mitis* S. Moore	Loganiaceae	T	Sidaamoo
*Syzygium guineense* (Willd.) DC	Myrtaceae	T	Baddeessa
*Terminalia brownii* Fresen	Combretaceae	T	Birdheessa
*Terminalia laxiflora* Engl. & Diels	Combretaceae	S	Dabaqqaa
*Withania somnifera* L. Dunal in DC	Solanaceae	T	Unzoo
*Ximenia americana* L	Olacaceae	S	Kuuloo/ Hudhaa
*Zanthoxylum chalybeum* Engl	Rutaceae	T	Gadda

10,

**Table 11:** List of plant species and parts used (PU), method of preparation (MPPn.), administration, use category (UC), area of applications (AAPs), Ip (number of informants mentioned for a particular use) Iu (number of informants mentioned for any use, fidelity level in percentages (FL%), total number of cited (TNuC), and ratio of number of use cited (NuC) in the study area

Species	PU	MPPn	Administration	UC	AAP	Ip	Iu	FL(%)	TNuC	NuC
*Acacia brevispica* Harms	Leaves	Decoction	The leaves are applied topically via sprinkling on wounds and pimples	Perfume	Skin	6	9	66.67	15	1.20
*Acacia drepanolobium* Harms ex Sjöstedt	Fruit	Maceration	The oil is extracted from the Fruit and applied topically to repair and beautify the skin	Skincare	Skin	7	10	70.00	17	1.36
*Acacia etbaica* Schweinf	Wood	Directly used	The trunk for brushing teeth	Teeth Hygiene	Teeth	7	12	58.33	19	1.52
*Acacia mellifera* (Vahl) Benth	Flowers	Maceration	The oil is extracted from the Flowers and applied topically Hair cream	Hair coloring	Hair	7	11	63.64	18	1.44
*Aloe citrina* Carter & Brandham	Whole plant	squeezing	The squeeze its body and use the fluid that comes out	Skincare	Skin	22	23	95.65	45	3.60
*Azadirachta indica* A. Juss	leaves	Maceration	Boiling the fresh leaves	Face cleaner	Face	8	9	88.89	17	1.36
*Boswellia neglecta* S.Moore	resin	Smoked	Resins are dropped on fire	Perfume	Skin	14	16	87.50	30	2.40
*Cadaba longifolia (*R. Br.) DC	Bark	Smoked	Pieces of Barks are burned on the fire	Perfume	Skin	6	9	66.67	15	1.20
*Carica papaya* L	Leaves	Decoction	The bark is applied topically on burn wounds (burns)	Skincare	Skin	10	12	83.33	22	1.76
*Citrus aurantifolia* (Christm.) Swingle	Bark	Maceration	The bark is applied topically on burn wounds (burns)	Face cleaner	Face	9	11	81.82	20	1.60
*Combretum adenogonium* Steud. ex A. Rich	Leaves	Decoction	The leaves are applied topically on wounds and sores	Face cleaner	Face	20	21	95.24	41	3.28
*Commiphora baluensis* Engl	Bark	Maceration	The bark is used for wounds and it rejuvenates the skin. Furthermore, it is applied topically on the skin or wounds	Face mask	Face	13	14	92.86	27	2.16
*Commiphora habessinica* (Berg) Engl	Leaves	Maceration	Leaves are applied topically on wounds	Hair health	Hair	16	17	94.12	33	2.64
*Commiphora myrrha* (Nees) Engl	Bark	Smoked	The bark is burned and the vagina is fumigated	Vaginal health	Vagina	8	11	72.73	19	1.52
*Cordia africana* Lam	Bark	Maceration	Barks are grinded and mixed with water	Hand decorative	Hand	8	9	88.89	17	1.36
*Cordia monoica* Roxb	Leaves	Decoction a	The leaves are used to wash the vagina	Vaginal health	Vagina	15	17	88.24	32	2.56
*Croton dichogamus* Pax	Bark	Maceration	The oil is extracted from the Bark and applied topically to repair and beautify the skin	Face mask	Face	9	11	81.82	20	1.60
*Croton macrostachyus* Del	Seeds	Maceration	Stimulate hair growth; it is applied topically	Hair cream	Hair	11	13	84.62	24	1.92
*Dalbergia commiphoroides* Bak. f	Leaves	Decoction	The bark is grinded and used	Skincare	skin	12	15	80.00	27	2.16
*Delonix elata* (L.) Gamble	Leaves	Decoction	The leaf is crushed and rubbed on the skin	Skincare	Skin	12	14	85.71	26	2.08
*Dobera glabra* (Forssk.) Poir	Roots	Maceration	Roots are applied topically and orally for mouth sores and as toothpaste	Teeth Hygiene	Teeth	6	8	75.00	14	1.12
*Dodonaea angustifolia* L. f	Wood	Directly used	The trunk is for brushing	Teeth Hygiene	Teeth	15	17	88.24	32	2.56
*Ehretia cymosa* Thonn	leaves	Maceration	Leaves are applied topically on wounds	Hand decorative	Hand	13	15	86.67	28	2.24
*Euphorbia tirucalli* L	Leaves	Directly used	Leaves are applied orally and as lotion on burned skin (burns) and wounds	Perfume	Skin	19	20	95.00	39	3.12
*Gnidia stenophylla* Gilg	Leaves	Maceration	The leaves are burned and applied topically on the wound	Skincare	Skin	15	16	93.75	31	2.48
*Grewia bicolor* Juss	Bark	Maceration	grinded and mixed with water	Face mask	Face	8	10	80.00	18	1.44
*Juniperus procera* Hochst. ex Endl	Wood	Smoked	By frightening his trunk	Perfume	Skin	5	8	62.50	13	1.04
*Kalanchoe lanceolata* Forssk.) Pers	Whole plant	Decoction	Grinding of root and bark	Skincare	Skin	7	10	70.00	17	1.36
*Kirkia burgeri* Stannard	Roots	Maceration	The oil is extracted from the root and applied topically Hair cream	Skincare	Skin	8	11	72.73	19	1.52
*Mimusops kummel* A. DC	Leaves	Decoction	The sap from the plant is squeezed directly on skin wounds	Skincare	Skin	7	11	63.64	18	1.44
*Murdannia simplex* (Vahl) Brenan	Roots	smoked	It is taken orally as a mouthwash	Teeth Hygiene	Teeth	15	16	93.75	31	2.48
*Olea europaea* L. subsp*. cuspidata* (Wall. ex G.Don)	Seeds	Maceration	Stimulate hair growth; it is applied topically	Hair cream	Hair	22	23	95.65	45	3.60
*Osyris quadripartita* Decn	Bark	Maceration	Bark is taken orally because it is believed the skin is affected from the inside	Hair health	Hair	9	11	81.82	20	1.60
*Persea americana* Mill	Leaves	Directly used	Leaves are applied topically on wounds	Face mask	Face	8	10	80.00	18	1.44
*Premna schimperi* Engl	Leaves	Decoction	Put the medicine on the infected teeth	Teeth Hygiene	Teeth	16	17	94.12	33	2.64
*Rhamnus staddo* A. Rich	leaves	Decoction	crushed the leaves and applied them	Perfume	Skin	8	11	72.73	19	1.52
*Rhus natalensis* Krauss	Wood	Directly used	A piece of the trunk is cut and used	Teeth Hygiene	Teeth	20	22	90.91	42	3.36
*Sesamothamnus rivae* Engl	Bark	smoked	The powder from the bark is applied as a paste on the mouth sores	Skincare	Skin	22	23	95.65	45	3.60
*Solanum hastifolium* Hochst. ex Dunal in DC	Roots	Decoction	The cream is applied on the skin(acne)	Skincare	Skin	6	9	66.67	15	1.20
*Solanum lycopersicum* L	Fruit	Squeezed	Fruit sap is administered topically as a facial wash	Face cleaner	Face	6	10	60.00	16	1.28
*Stereospermum kunthianum* Cham	Bark	Directly used	A piece of bark is cut and used	Teeth Hygiene	Teeth	16	18	88.89	34	2.72
*Strychnos mitis* S. Moore	Wood	Smoked	A piece of wood is burned on a small fire	Skincare	Skin	7	9	77.78	16	1.28
*Syzygium guineense* (Willd.) DC	Bark	Smoked	A piece of bark is burned on a small fire	Perfume	Skin	21	22	95.45	43	3.44
*Terminalia brownii* Fresen	Seeds	Maceration	Mixed with soil for cleaning teeth. It is used orally as toothpaste	Hair cream	Hair	23	24	95.83	47	3.76
*Terminalia laxiflora* Engl. & Diels	Bark	Maceration	It is applied topically on wounds	Skincare	Skin	22	23	95.65	45	3.60
*Withania somnifera* L. Dunal in DC	Wood	Decoction	leaves	Perfume	Skin	7	10	70.00	17	1.36
*Ximenia americana* L	Wood	Directly used	The trunk for brushing teeth	Teeth Hygiene	Teeth	14	16	87.50	30	2.40
*Zanthoxylum chalybeum* Engl	Bark	Squeezed	squeezed his cock and applied the fluid	Hair coloring	Hair	9	11	81.82	20	1.60

^11^,

**Table 12:** Relative frequency of citation and cultural importance index of plant species used in a traditional cosmetic among the Oromo Women in Madda Walabu District, Bale Zone, Southeast Ethiopia

Scientific name	Relative frequency of citation	Cultural importance index
*Acacia brevispica*	0.04	0.10
*Acacia drepanolobium*	0.05	0.11
*Acacia etbaica*	0.05	0.13
*Acacia mellifera*	0.05	0.12
*Aloe citrina*	0.15	0.30
*Azadirachta indica*	0.05	0.11
*Boswelia neglecta*	0.09	0.20
*Cadaba longifolia*	0.04	0.10
*Carica papaya*	0.07	0.15
*Citrus aurantifolia*	0.06	0.13
*Combretum adenogonium*	0.13	0.27
*Commiphora baluensis*	0.09	0.18
*Commiphora habessinica*	0.11	0.22
*Commiphora myrrha*	0.05	0.13
*Cordia africana*	0.05	0.11
*Cordia monoica*	0.10	0.21
*Croton dichogamus*	0.06	0.13
*Croton macrostachyus*	0.07	0.16
*Dalbergia commiphoroides*	0.08	0.18
*Delonix elata*	0.08	0.17
*Dobera glabra*	0.04	0.09
*Dodonaea viscosa*	0.10	0.21
*Ehretia cymosa*	0.09	0.19
*Euphorbia tirucalli*	0.13	0.26
*Gnidia stenophylla*	0.10	0.21
*Grewia bicolor*	0.05	0.12
*Juniperus procera*	0.03	0.09
*Kalanchoe lanceolata*	0.05	0.11
*Kirkia burgeri Stannard subsp.*	0.05	0.13
*Mimusops kummel*	0.05	0.12
*Murdannia simplex*	0.10	0.21
*Olea europaea L. subsp.cuspidata*	0.15	0.30
*Osyris quadripartita*	0.06	0.13
*Persea Americana*	0.05	0.12
*Premna schimperi*	0.11	0.22
*Rhamnus staddo*	0.05	0.13
*Rhus natalensis*	0.13	0.28
*Sesamothamnus rivae*	0.15	0.30
*Solanum hastifolium*	0.04	0.10
*Solonum lycopersicum*	0.04	0.11
*Stereospermum kunthianum*	0.11	0.23
*Strochynos mitis*	0.05	0.11
*Syzygium guineense*	0.14	0.29
*Terminalia brownii*	0.15	0.31
*Terminalia laxiflora*	0.15	0.30
*Withania somnifera*	0.05	0.11
*Ximenia Americana*	0.09	0.20
*Zanthoxylum chalybeum*	0.06	0.13

^12^ and

**Table 13:** Names, coordinates, and altitudes of visited locations to collect data on the ethnobotany of traditional cosmetics among the Oromo women in the selected three villages of Madda Walabu district, Bale Zone, Southeast Ethiopia

Sites	Longitude (degrees, minutes and seconds)	Latitude (degrees, minutes and seconds)	Altitude (m asl.)
Aba Sirba	55,614	394,812	1211
Aba Sirba	55,614	394,810	1223
Aba Sirba	55,556	394,717	1349
Aba Sirba	55,519	394,644	1284
Aba Sirba	55,556	394,914	1152
Aba Sirba	55,521	394,821	815
Aba Sirba	55,631	394,818	1196
Aba Sirba	55,630	394,814	1201
Aba Sirba	55,636	394,809	1211
Aba Sirba	55,637	394,809	1212
Aba Sirba	55,636	394,808	1210
Aba Sirba	55,638	394,806	1211
Aba Sirba	55,640	394,755	1223
Aba Sirba	55,642	394,752	1225
Aba Sirba	55,644	394,752	1226
Aba Sirba	55,641	394,748	1120
Aba Sirba	55,628	394,949	1121
Aba Sirba	55,717	394,916	1372
Aba Sirba	55,707	395,023	1211
Aba Sirba	55,724	395,016	1211
Aba Sirba	55,748	395,030	1211
Aba Sirba	55,605	395,125	1154
Aba Sirba	55,602	394,953	1156
Aba Sirba	55,601	394,952	1154
Aba Sirba	55,544	394,922	1156
Aba Sirba	55,545	394,923	1154
Aba Sirba	55,548	394,950	1147
Aba Sirba	55,511	394,914	1276
Aba Sirba	55,711	395,051	1112
Aba Sirba	55,650	395,024	1099
Aba Sirba	55,650	395,023	1098
Aba Sirba	55,237	394,429	1591
Aba Sirba	55,232	394,444	1571
Aba Sirba	55,232	394,433	1587
Aba Sirba	55,227	394,445	1574
Aba Sirba	55,223	394,441	1584
Aba Sirba	55,327	394,418	1602
Aba Sirba	55,326	394,417	1600
Aba Sirba	53,228	394,421	1598
Aba Sirba	55,225	394,415	1605
Aba Sirba	55,225	394,416	1604
Aba Sirba	55,229	394,419	1601
Aba Sirba	55,229	394,420	1598
Aba Sirba	55,233	394,413	1599
Aba Sirba	55,237	394,413	1592
Aba Sirba	55,239	394,415	1589
Aba Sirba	55,224	394,416	1604
Aba Sirba	55,222	394,417	1606
Aba Sirba	55,220	394,419	1612
Aba Sirba	55,217	394,422	1611
Hora Kore	60,500	394,501	975
Hora Kore	60,503	394,448	963
Hora Kore	60,459	39,447	960
Hora Kore	60,454	39,441	965
Hora Kore	60,452	394,432	966
Hora Kore	60,457	394,437	972
Hora Kore	60,453	394,438	970
Hora Kore	60,506	394,434	977
Hora Kore	60,508	394,436	972
Hora Kore	60,508	394,421	978
Hora Kore	60,503	394,424	973
Hora Kore	60,500	394,425	970
Hora Kore	60,500	394,423	970
Hora Kore	60,457	394,425	965
Hora Kore	60,458	394,428	972
Hora Kore	60,450	394,428	959
Hora Kore	60,448	394,431	961
Hora Kore	60,452	394,430	965
Hora Kore	60,452	394,432	966
Hora Kore	60,501	394,437	972
Hora Kore	60,459	394,441	972
Hora Kore	60,455	394,443	964
Hora Kore	60,514	394,426	991
Hora Kore	60,448	394,433	960
Hora Kore	60,454	394,437	967
Hora Kore	60,452	394,425	960
Hora Kore	60,510	394,428	986
Hora Kore	60,514	394,430	985
Hora Kore	60,509	394,430	983
Hora Kore	60,506	394,431	982
Hora Kore	60,506	394,431	980
Hora Kore	60,509	394,425	985
Hora Kore	60,508	394,426	983
Hora Kore	60,508	394,428	983
Hora Kore	60,506	394,426	979
Hora Kore	60,456	394,433	970
Hora Kore	60,456	394,434	972
Hora Kore	60,457	394,435	973
Hora Kore	60,458	394,436	973
Hora Kore	60,456	394,436	972
Hora Kore	60,458	394,438	970
Hora Kore	60,458	394,438	969
Hora Kore	60,457	394,440	967
Hora Kore	60,456	394,441	966
Hora Kore	60,455	39,441	965
Hora Kore	60,456	394,439	968
Hora Kore	60,456	394,440	967
Hora Kore	60,456	394,440	966
Hora Kore	60,454	394,441	966
Hora Kore	60,500	394,428	972
Hora Kore	60,501	394,427	972
Hora Kore	60,502	394,426	972
Hora Kore	60,456	394,429	967
Hora Kore	60,454	394,428	965
Hora Kore	60,458	394,431	974
Hora Kore	60,459	394,423	964
Hora Kore	60,458	394,424	963
Hora Kore	60,458	394,424	963
Hora Kore	60,456	394,431	970
Hora Kore	60,458	394,433	974
Medacho	55,203	393,504	1222
Medacho	55,156	393,506	1213
Medacho	55,150	393,507	1219
Medacho	55,128	393,504	1288
Medacho	55,146	393,426	1252
Medacho	55,140	3,993,429	1253
Medacho	55,136	393,432	1251
Medacho	55,154	393,256	1284
Medacho	55,121	393,449	1261
Medacho	55,115	393,453	1258
Medacho	55,106	393,450	1275
Medacho	55,105	393,503	1246
Medacho	55,057	393,454	1258
Medacho	55,216	393,336	1289
Medacho	55,100	393,522	1243
Medacho	55,115	393,439	1281
Medacho	55,237	393,419	1243
Medacho	55,207	393,153	1277
Medacho	55,238	393,409	1238
Medacho	55,237	393,412	1237
Medacho	55,238	393,403	1242
Medacho	55,213	393,420	1247
Medacho	55,236	393,422	1258
Medacho	55,224	393,401	1262
Medacho	55,216	393,405	1253
Medacho	55,210	393,414	1252
Medacho	55,228	393,504	1269
Medacho	55,252	393,419	1263
Medacho	55,301	393,404	1260
Medacho	55,311	393,348	1265
Medacho	55,255	393,437	1340
Medacho	55,223	393,510	1247
Medacho	55,151	393,412	1261
Medacho	55,247	393,454	1380
Medacho	55,217	393,459	1248
Medacho	55,213	393,517	1231
Medacho	55,203	393,424	1237
Medacho	55,143	393,518	1212
Medacho	55,204	393,524	1227
Medacho	55,156	393,530	1224

^13^.

## Abbreviations

ANOVA: Analysis of variance
CI: Cultural importance index
FL: Fidelity level
ICF: Informant consensus factor
RFC: Relative frequency of citations

## References

[1] Gebelein CG (1997). Chemistry and our world.

[2] Shaheen H, Nazir J, Firdous SS (2014). Cosmetic ethnobotany practiced by tribal women of Kashmir Himalayas. Avicenna J Phytomed.

[3] McMullen RL, Dell’Acqua G (2023). History of natural ingredients in cosmetics. Cosmetics.

[4] Manniche L (1989). An ancient Egyptian herbal.

[5] El-Demerdash M, Saxena PK (2001). Medicinal plants of Egypt. Development of plant-based medicines: conservation efficacy and safety.

[6] Blanco-Dávila F (2000). Beauty and the body: the origins of cosmetics. Plast Reconst Surg.

[7] Allied market research. Global cosmetics market (category, mode of sale, gender, and geography) size. Share, trends, company profiles, demand, analysis, growth, opportunities, and forecast 2014–2020. Portland, Oregon. 2015.

[8] Gediya SK, Mistry RB, Patel UK, Blessy M, Jain HN (2011). Herbal plants: used as cosmetics. J Nat Prod Plant Resour.

[9] UNEP. Green economy sectorial l study: biotrade—a catalyst for transitioning to a green economy in Namibia 2012; 35–64. https://wedocs.unep.org/20.500.11822/25957.

[10] Mafra AL, Varella MAC, Defelipe RP, Anchieta NM, de Almeida CAG, Valentova JV (2020). Makeup usage in women as a tactic to attract mates and compete with rivals. Pers Individ Differ.

[11] Pfeiffer JM, Butz RJ (2015). Assessing cultural and ecological variation in ethnobiological research: the importance of gender. J Ethnobiol.

[12] Abebe T, Mulu D (2017). The role of women in the management and utilization of home garden: the case of Dale district, in southern Ethiopia. Asian J Plant Sci Res.

[13] Aseffa W, Kawessa G, Datiko D (2022). Agrobiodiversity and gender: the role of women in farm diversification among smallholder farmers in Sinana district. Southeast Ethiop Biodivers Conserv.

[14] Newman DJ, Cragg GM (2012). Natural products as sources of new drugs over the 30 years from 1981 to 2010. J Nat Prod.

[15] Howard PL (2003). Women and plants: gender relations in biodiversity management and conservation.

[16] Bilal A, Tilahun Z, Shimels T, Gelan YB, Osman ED (2016). Cosmetics utilization practice in Jigjiga town, eastern Ethiopia: a community-based cross-sectional study. Cosmetics.

[17] Abdurhman N. Plant diversity, ethnobotany, and barcoding of medicinal and cosmetic plants in Kalu and Bati districts of Amhara region, Ethiopia. Doctoral dissertation, Addis Ababa University. Addis Ababa, 2020.

[18] CSA, Ethiopian central statistical service. Projected population of districts in Ethiopia. Addis Ababa, Ethiopia, 2023.

[19] NMAE, National meteorological agency of Ethiopia. Climate data, Bale Robe meteriological sub-station. Bale Robe, Ethiopia, 2022.

[20] Madda Walabu district agricultural office (MWDAO). Socio-economic base line survey. Madda Walabu, Bale Zone, Oromia Regional State, Ethiopia, 2023.

[21] Friis I, Demissew S, van Breugel P (2010). The atlas of potential vegetation of Ethiopia.

[22] Legesse A (2006). Oromo democracy: an indigenous african political system.

[23] UNESCO. Intangible cultural heritage. Gada system, an indigenous democratic socio-political system of the Oromo.ich.unesco.org. 2016.

[24] Hinew D (2012). History of oromo social organization: gadaa grades based roles and responsibilities science. Technol Art Res J.

[25] Jalata A (2012). Gada (oromo democracy): an example of classical African civilization. J PAS.

[26] Boku DD (2011). Oromo wisdom in black civilization.

[27] Dibaba AT (2020). Oromo orature: an ecopoetic approach, theory and practice (Oromia/Ethiopia, northeast Africa). Humanities.

[28] Tongco MDC (2007). Purposive sampling as a tool for informant selection. Ethnobot Res App.

[29] Plants of the world. https://powo.science.kew.org. Accessed on 15 Feb 2024.

[30] Hoffman B, Gallaher T (2007). Importance indices in ethnobotany. Ethnobot Res Appl.

[31] R Core Team. R: a language and environment for statistical computing. R Foundation for Statistical Computing, Vienna, Austria. ISBN 3–900051–07–0. http://www.R-project.org/ (accessed 30 Oct 2022).

[32] Heinrich MA, Ankli B, Frei C, Weimann C, Sticher O (1998). Medicinal plants in Mexico: Healer’s consensus and cultural importance. Soc Sci Med.

[33] Tardio J, Pardo-de-Santayana M (2008). Cultural importance indices: a comparative analysis based on the useful wild plants of southern Cantabria (northern Spain) 1. Econ Bot.

[34] Saikia AP, Ryakala VK, Sharma P, Goswami P, Bora U (2006). Ethnobotany of medicinal plants used by Assamese people for various skin ailments and cosmetics. J Ethnopharmacol.

[35] Abbasi AM, Khan MA, Ahmad M, Zafar M, Jahan S, Sultana S (2010). Ethnopharmacological application of medicinal plants to cure skin diseases and in folk cosmetics among the tribal communities of north–west Frontier province. Pak J Ethnopharmacol.

[36] Khabbach A, Libiad M, Ennabili A, Bousta D (2012). Medicinal and cosmetic use of plants from the province of Taza, Northern Morocco. Bol Latinoam Caribe Plantas Med Aromat.

[37] Gamage DGND, Dharmadasa RM, Abeysinghe DC, Wijesekara RGS, Gamika A, Prathapasinghe GA, Someya T (2022). Global perspective of plant-based cosmetic industry and possible contribution of Sri Lanka to the development of herbal cosmetics. Evid Complement Alternat Med.

[38] Kohli K, Ahmed N, Baboota S (2004). Herbs in cosmetics. Hamdard Med.

[39] Ndhlovu PT, Mooki O, Mbeng WO, Aremu AO (2019). Plant species used for cosmetic and cosmeceutical purposes by the Vhavenda women in Vhembe district municipality, Limpopo. S Afr J Bot.

[40] Elansary HO, Mahmoud EA, Shokralla S, Yessoufou K (2015). Diversity of plants, traditional knowledge, and practices in local cosmetics: a case study from Alexandria. Egypt Econ Bot.

[41] Fedoung EF, Zra T, Biyegue CFN, Bissoue AN, Bauye S, Tsabeng N (2018). Herbal cosmetics knowledge of Arab-Choa and Kotoko ethnic groups in the semi-arid areas of Far North Cameroon: ethnobotanical assessment and phytochemical review. Cosmetics.

[42] Mahomoodally FM, Ramjuttun P (2016). A quantitative ethnobotanical survey of phytocosmetics used in the tropical island of Mauritius. J Ethnopharmacol.

[43] Mwinga JL, Makhaga NS, Aremu AO, Otang-Mbeng W (2019). Botanicals used for cosmetic purposes by Xhosa women in the Eastern Cape South Africa. S Afr J Bot.

[44] Didita M, Nemomissa S, Gole TM (2010). Floristic and structural analysis of the woodland vegetation around Dello Menna, southeast Ethiopia. J For Res.

[45] Asfaw MM, Abebe FB (2021). Traditional medicinal plant species belonging to Fabaceae family in Ethiopia: a systematic review. Int J Plant Biol.

[46] Leulekal E, Kelbessa E, Bekele T, Yineger H (2008). An ethnobotanical study of medicinal plants in Mana Angetu district, southeastern Ethiopia. J Ethnobiol Ethnomed.

[47] Assefa B, Megersa M, Tolossa T (2021). Ethnobotanical study of medicinal plants used to treat human diseases in Gura Damole district, Bale Zone, and southeast Ethiopia. Asian J Ethnobiol.

[48] Peter KV, Peter KV (2012). Introduction to herbs and spices: definition, trade and applications. Handbook of herbs and spices.

[49] Gazzaneo LRS, Lucena RFP, Albuquerque UP (2005). Knowledge and use of medicinal plants by local specialists in a region of Atlantic forest in the state of Pernambuco (Northeastern Brazil). J Ethnobiol Ethnomed.

[50] Afolayan AJ, Grierson DS, Mbeng WO (2014). Ethnobotanical survey of medicinal plants used in the management of skin disorders among the Xhosa communities of the Amathole district, Eastern Cape. South Africa J Pharmacol.

[51] Fongnzossie EF, Tize Z, Fogang Nde PJ, Nyangono Biyegue CF, Bouelet Ntsama IS, Dibong SD, Nkongmeneck BA (2017). Ethnobotany and pharmacognostic perspective of plant species used as traditional cosmetics and cosmeceuticals among the Gbaya ethnic group in Eastern Cameroon. S Afr J Bot.

[52] Edwards S, Tadesse M and Hedberg I. Flora of Ethiopia and Eritrea volume 2, part 2. Addis Ababa, Ethiopia. 1995; pp. 115–132.

[53] Okeyo MM, Obwoyere GO, Makanji DL, Njuguna JW, Atieno J (2020). Promotion of Terminalia brownii in reforestation by development of appropriate dormancy breaking and germination methods in drylands; Kenya. Glob Ecol Conserv.

[54] Mariod AA, Mohammed NM, Nabag FO, Hassan AA (2014). Ethnobotanical study of three trees: indigenous knowledge on trees used as cosmetic in Khartoum state Sudan. AJPS.

[55] Sbhatu DB, Berhe GG, Hndeya AG, Abdu A, Mulugeta A, Abraha HB, Weldemichael MY, Tekle HT, Gebru HA, Taye MG, Kidanemariam GH (2020). Hair washing formulations from aloe elegans todaro gel: the potential for making hair shampoo. Adv Pharmacol Pharm Sci.

[56] Oda BK, Erena BA (2017). Aloes of Ethiopia: a review on uses and importance of aloes in Ethiopia. Int J Plant Biol.

[57] Kaur A, Singh TG, Dhiman S, Arora S, Babbar R (2020). Novel herbs used in cosmetics for skin and hair care: a review. Plant Arch.

[58] Asfaw A, Lulekal E, Bekele T, Debella A, Debebe E, Sisay B (2022). Medicinal plants used to treat livestock ailments in Ensaro district, North Shewa zone, Amhara regional state. Ethiopia BMC Vet Res.

[59] Luizza MW, Young H, Kuroiwa C, Evangelista P, Worede A, Bussmann RW, Weimer A (2013). Local knowledge of plants and their uses among women in the Bale Mountains Ethiopia. Ethnobot Res Appl.

[60] Khongsai M, Saikia SP, Kayang H (2011). Ethnomedicinal plants used by different tribes of Arunachal Pradesh. I J T K.

[61] Jan G, Khan MA, Gul F (2009). Ethnomedicinal plants use against jaundice in Dir Kohistan valleys (NWFP). Pak Ethnobot Leafl.

[62] Hemminki E, Mantyranta T, Malin M, Koponen P (1991). A survey on the use of alternative drugs during pregnancy. Scand J Soc Med.

[63] Alencar NL, Junior WSF, Albuquerque UP (2014). Medicinal plant knowledge richness and sharing in Northeastern Brazil. Econ Bot.

